# Novel and diverse features identified in the genomes of bacteria isolated from a hydrothermal vent plume

**DOI:** 10.1128/aem.02593-24

**Published:** 2025-03-31

**Authors:** S. R. Major, J. M. Polinski, K. Penn, M. Rodrigue, M. J. Harke

**Affiliations:** 1 Gloucester Marine Genomics Institute 590683, Gloucester, Massachusetts, USA; 2 OceanX, New York, New York, USA; University of Delaware, Lewes, Delaware, USA

**Keywords:** hydrothermal vent plume, bacteria, genome, microbial ecology, biosynthetic gene cluster

## Abstract

Hydrothermal vent plumes (HVPs), formed by high-temperature vent emissions, are rich in compounds that support chemosynthesis and serve as reservoirs of microbial diversity and genetic innovation. Through turbulence, mixing, and interaction with subsea currents, vent communities are thought to disperse across ocean basins. In this study, we focused on the plume of the Moytirra hydrothermal vent field, a relatively unexplored site, to investigate its microbial inhabitants. We cultured bacteria from the Moytirra HVP using 11 different media types and performed complete genome sequencing on 12 isolates. Our analyses revealed four putatively novel species from the *Thalassobaculum*, *Sulfitobacter*, *Idiomarina*, and *Christiangramia* genera. Comparative genomics identified unique genomic islands containing biosynthetic gene clusters, including a novel Non-Ribosomal Peptide Synthetase/Polyketide Synthase cluster, toxin-antitoxin systems, and evidence of horizontal gene transfer facilitated by prophages. These findings underscore the potential of HVPs as a source of novel microbial species and biotechnologically relevant genes, contributing to our understanding of the biodiversity and genetic complexity of these extreme environments.

**IMPORTANCE:**

Hydrothermal vents are dynamic environments that offer unique nutrients for chemosynthetic organisms to drive biology in the deep-sea. The dynamics of these ecosystems are thought to drive genomic innovation in resident populations. Hydrothermal vent plumes (HVPs) mix with surrounding water, carrying local microbiota with them and dispersing for hundreds of kilometers. This study isolated bacteria from a HVP to capture a genomic snapshot of the microbial community, revealing four putatively novel species of bacteria within three taxonomic classes. The addition of these genomes to public databases provides valuable insights into the genomic function, architecture, and novel biosynthetic gene clusters of bacteria found in these extreme environments.

## INTRODUCTION

Hydrothermal vent plumes (HVPs) are distinct marine environments flowing from hydrothermal vents, which can act as underwater highways that disperse microbial communities across ocean basins for hundreds of kilometers or more (1, 2). HVPs are characterized by dynamic gradients of temperature and chemistry, ranging from hot (>350°C) anoxic hydrothermal fluids to cold (~4°C), oxygenated seawater, and can support chemosynthetic ecosystems driven by compounds like H_2_S, Fe, Mn, CH_4_, and H_2_ (1, 3, 4). Although influenced by ambient seawater, HVPs contain organisms from vent fauna, bottom water, and the subsurface (5–7). Bacteria dispersed within the plume are further affected by phages carrying genes that may enhance host fitness, suggesting that horizontal gene transfer (HGT) plays a key role in spreading genes related to energy metabolism and secondary metabolites (1). Likewise, horizontally acquired genomic islands (GIs) have also been recognized as areas indicative of trophic specialization (8) and suggested to be genetically dynamic regions that naturally develop secondary metabolite diversity (9). HVPs are thought to be reservoirs of genomic innovation (10, 11) and are invaluable sources for natural product discovery (12–14).

Due to difficulties applying culture-dependent techniques to deep-sea bacteria, most work on hydrothermal vent microbial communities has been culture-independent via ‘omic methods such as metagenomics and metatranscriptomics. Culture-independent research on the Moytirra HVP using metatranscriptomics highlighted the importance of these approaches for understanding microbial community dynamics (15). However, culture-dependent methods complement these by enabling detailed investigations into the metabolism and functions of specific community members (16, 17). A recent study underscored the complementary nature of these methods by revealing that culture-independent assessments of microbial communities in marine sediments missed 39 genera that culture-dependent efforts successfully identified (18). This emphasizes the value of both methods in achieving a more comprehensive understanding of microbial communities.

The Moytirra hydrothermal vent field is a high-temperature deep-sea site discovered in 2011 (19) and has been described in terms of geology and captured macrofauna (19, 20) as well as more recently by evaluating vent plume biodiversity (metabarcoding) and function (metatranscriptome) across a spatial transect (15). The current study presents whole-genome sequencing (WGS) of 12 bacteria isolated from the Moytirra HVP complemented with metatranscriptomic and 16S community data (15). The complete genome assembly of local bacterial residents sheds light on their functional attributes and identifies four putatively novel species and the potential function of unique genomic regions known as GIs.

## RESULTS

One liter of water was collected from the Moytirra HVP, filtered to capture cells on a 0.22 µm filter, and cryopreserved until plated for culturing and isolation. Over a period of 7–28 days, 173 bacteria were isolated and assigned an “OXR” identifier (OXR = OceanX Research). Twelve isolates were selected for WGS based on several criteria including 16S rRNA gene novelty, presence in Moytirra HVP community 16S data, and relevance to the HVP environment (Table 2; Table S2).

### Genome assembly and taxonomy

Six assemblers used Illumina and Oxford Nanopore Technologies (ONT) WGS data to generate preliminary assemblies used as input in Trycycler, resulting in 12 bacterial genomes, all exhibiting >98% completeness scores. Assemblies comprised one to eight circular contigs, indicating the presence of a primary chromosome and extrachromosomal plasmids (Table 1; Table S3).

**TABLE 1:** Assembly summary statistics of each of the 12 isolates^*a*^

Taxonomy	Contigs	Size (Mb)	GC%	Predicted genes	ONT coverage	BUSCO	CHECKM
*Sulfitobacter faviae* OXR-9	8	3.96	62.3	3,856	68×	100.0%	99.7%/0.2%
*Roseivirga spongicola* OXR-11	1	4.48	40.2	3,957	35×	99.2%	99.1%/0.0%
*Geobacillus subterraneus* OXR-76	1	3.54	52.2	3,496	411×	99.2%	99.4%/0.0%
*Nitratireductor rhodophyticola* OXR-85	3	4.68	61.5	4,430	139×	100.0%	100.0%/1.7%
*Pelagerythrobacter marinus* OXR-96	1	3.32	67.5	3,175	117×	98.4%	99.5%/0.3%
*Blastomonas marina* OXR-134	1	2.84	65.0	2,738	166×	99.2%	99.1%/0.2%
*Thalassobaculum* sp. OXR-137*	3	5.65	67.0	5,146	114×	100.0%	100.0%/0.4%
*Sulfitobacter* sp. OXR-159*	8	4.42	60.8	4,450	35×	100.0%	99.4%/0.3%
*Idiomarina* sp. OXR-189*	1	2.60	47.1	2,476	141×	99.2%	99.6%/0.3%
*Sulfitobacter pontiacus* OXR-199	7	4.04	60.2	3,880	122×	99.2%	99.4%/0.5%
*Christiangramia* sp. OXR-203*	1	3.40	38.4	3,109	109×	99.2%	99.6%/0.2%
*Limimaricola variabilis* OXR-209	7	4.22	67.6	4,072	93×	99.2%	99.2%/1.1%

^*a*^ Asterisks (*) denote putatively novel species determined by digital DNA-DNA hybridization (dDDH) values <70%, indicating a potential new species distinct from the TYGS database. Percentages are shown for BUSCO completeness and CHECKM results (completeness/contamination).

Phylogenetic and taxonomic analysis of the largest chromosome using Type (Strain) Genome Server (TYGS; 21) classified eight isolates to species level, and four isolates exhibited digital DNA-DNA hybridization (dDDH) values <70%, suggesting they may represent novel species (Table 2). dDDH values are provided in parentheses hereafter. Three phyla—Pseudomonadota, Bacteroidota, and Bacillota—were represented among the 12 isolates. The Alphaproteobacteria (Pseudomonadota) has two putative new species *Sulfitobacter* sp. OXR-159 (67.6%) and *Thalassobaculum* sp. OXR-137 (33.2%). It also has the isolates *Nitratireductor rhodophyticola* OXR-85 (81.7%), *Sulfitobacter faviae* OXR-9 (76%), *Sulfitobacter pontiacus* OXR-199 (79.1%), *Limimaricola variabilis* OXR-209 (75.9%), *Pelagerythrobacter marinus* OXR-96 (87.1%), and *Blastomonas marina* OXR-134 (84.3%). The Gammaproteobacteria (Pseudomonadota) has the putative new species *Idiomarina* sp. OXR-189 (68.8%), while Bacteroidota has the putative new species *Christiangramia* sp. OXR-203 (28.1%), and the isolate *Roseivirga spongicola* OXR-11 (91.1%). The Bacillota has the hyperthermophile *Geobacillus subterraneus* OXR-76 (85.8%; Table 2).

**TABLE 2:** Genome relatedness values of each isolate compared to their predicted type-strain^*a*^

Novel strain	Type strain	dDDH %	ANI %	16S %	16S match	dDDH %	ANI %	16S %
*Sulfitobacter faviae* OXR-9	*Sulfitobacter faviae* JCM 31093	76.0	97.1	99.9–100				
*Roseivirga spongicola* OXR-11	*Roseivirga spongicola* UST030701-084	91.1	99.0	100				
*Geobacillus subterraneus* OXR-76	*Geobacillus subterraneus* KCTC 3922	85.8	98.3	98.6–99.9				
*Nitratireductor rhodophyticola* OXR-85	*Nitratireductor rhodophyticola* L1-7-SE	81.7	98.1	99.4				
*Pelagerythrobacter marinus* OXR-96	*Pelagerythrobacter marinus* H32	87.1	98.5	100				
*Blastomonas marina* OXR-134	*Blastomonas marina* CGMCC 1.15297	84.3	98.1	100				
*Thalassobaculum* sp. OXR-137*	*Thalassobaculum litoreum* DSM 18839	33.2	89.3	99.8	*Thalassobaculum salexigens* DSM 19539	33.4	89.4	99.8
*Sulfitobacter* sp. OXR-159*	*Sulfitobacter indolifex* SAORIC-263	67.6	96.3	99.6	*Sulfitobacter dubius* CI.11.F.A3	36.3	90.1	99.9
*Idiomarina* sp. OXR-189*	*Idiomarina zobellii* KMM 231	68.8	96.7	98.1–100	*Idiomarina* sp. X4	31	87.8	98.8–99.1
*Sulfitobacter pontiacus* OXR-199	*Sulfitobacter pontiacus* DSM 10014	79.1	97.2	99.9				
*Christiangramia* sp. OXR-203*	*Christiangramia portivictoriae* DSM 23547	28.1	85.5	98.9–99.0	*Christiangramia echinicola* MAR_2010_102	19.4	80.1	98.0
*Limimaricola variabilis* OXR-209	*Limimaricola variabilis* CECT 8572	75.9	96.8	99.9–100				

^*a*^ Asterisks (*) denote putatively novel species determined by digital DNA-DNA hybridization (dDDH) values <70%.Putatively novel species are supplemented with genomic comparisons with a 16S rRNA gene match. Column 16S% ID is provided as a range when multiple copies of 16S rRNA genes exist.

Genome sizes ranged from 2.60 to 5.65 Mb (Table 1). Six Alphaproteobacteria contain two to seven plasmids, while those without plasmids are from the Sphingomonadales order (Table S3). The 16S, 23S, and 5S rRNA genes are primarily found on the largest chromosome of each isolate, but copies of the rRNA operon are also found on a plasmid of *S. pontiacus* OXR-199 (395 kb plasmid, pOXR199-01) and *L. variabilis* OXR-209 (51 kb plasmid, pOXR209-06) (Table S3). Although mapped paired-end Illumina reads to these regions displayed some improperly paired reads, Nanopore data mapped cleanly suggesting these regions were not chimeric and as such, misassembled. The Roseobacteraceae (OXR-9, OXR-159, OXR-199, and OXR-209), Bacteroidota (OXR-11 and OXR-203), *Idiomarina* sp. OXR-189, and *G. subterraneus* OXR-76 contain sequence variants among their multiple rRNA gene loci. Each genome encodes tRNAs for the 20 standard amino acids, with *S. pontiacus* OXR-199 and *P. marinus* OXR-96 additionally encoding tRNAs for selenocysteine and pyrrolysine, respectively.

### Functional annotations

**Fig 1:**
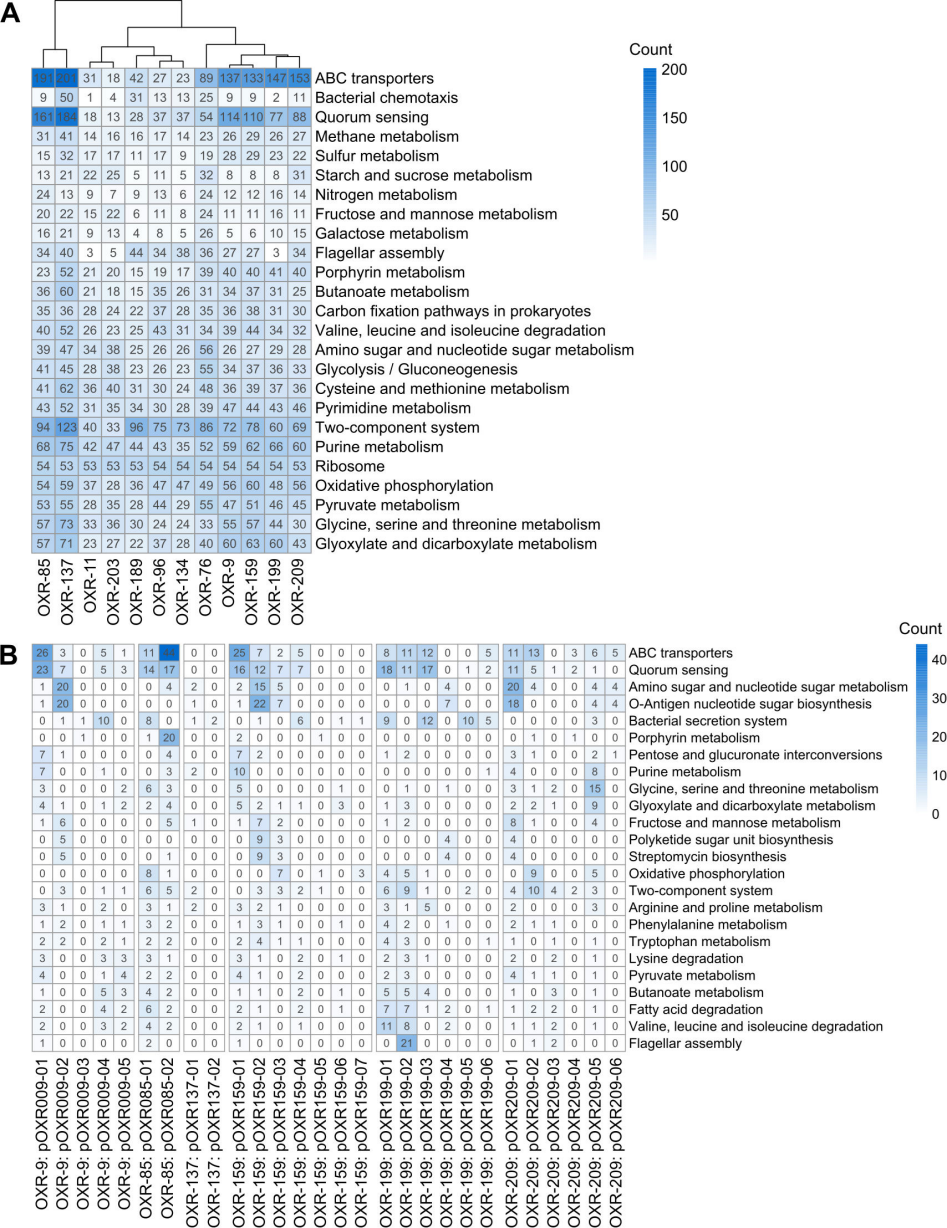
Annotated KEGG functions of (**A**) the primary chromosomes and (**B**) associated plasmids. Counts represent the total observed counts of unique KEGG ortholog IDs to the provided function.

The most abundant KEGG categories among the Alphaproteobacteria (OXR-9, OXR-85, OXR-96, OXR-134, OXR-137, OXR-159, OXR-199, and OXR-209) include ABC transporters (*n* > 130), quorum sensing components (*n* > 75), and two-component systems (*n* > 60), with *N. rhodophyticolas* OXR-85, *Thalassobaculum* sp. OXR-137 and members of the Roseobacteraceae (OXR-9, OXR-159, OXR-199, and OXR-209) show the highest counts (Fig. 1A; Table S4). In contrast, the Sphingomonadales (OXR-96 and OXR-134) exhibit fewer ABC transporters and quorum sensing genes compared to the other Alphaproteobacteria (Fig. 1A; Table S4). Sulfur metabolism and porphyrin biosynthesis genes (e.g., heme and B12 synthesis) are more prevalent in *Thalassobaculum* sp. OXR-137 and the Roseobacteraceae (*n* ≥ 40; Fig. 1A; Table S4). Flagellar assembly genes are common across most isolates, except for the two Bacteroidota (OXR-11 and OXR-203; Fig. 1A; Table S4). Isolates with more than 80 KEGG orthology terms to two-component system genes (*G. subterraneus* OXR-76, *Idiomarina* sp. OXR-189, and *Thalassobaculum* sp. OXR-137) also have a higher number of chemotaxis annotations (*n* = 25, 31, and 50, respectively), except for *N. rhodophyticola* OXR-85. *G. subterraneus* OXR-76 and *N. rhodophyticola* OXR-85 have the most annotations associated with nitrogen metabolism (*n* = 24; Fig. 1; Table S4).

### Motility

Motility is likely crucial in the plume environment due to the extreme chemical gradients that develop as the plume disperses from the hydrothermal vent (15). All isolates showed genetic evidence of motility (Table S5). The Pseudomonadota and Bacillota (all but OXR-11 and OXR-203) have genes necessary to build a motor and its associated structural proteins, along with the rod, hook, filament, and the type III secretion system proteins required to elongate the flagellar appendage (Table S5). Unlike the other bacteria, *S. pontiacus* OXR-199 carries its flagellar assembly genes on a plasmid (pOXR199-02; Fig. 1B). *Idiomarina* sp. OXR-189 shows evidence to code for a sodium-driven flagellar motor protein variant with genes *pomA*, *motX*, and *motY* (Table S5). The Roseobacteraceae were found to lack the stress response and flagellar regulatory gene *rpoN* (22), but it was found in all other isolates. The Bacillota does not carry the housekeeping gene *rpoD* that is found in the Pseudomonadota and Bacteroidota isolates but has the alternative sigma factors *sigA* and *sigD* for housekeeping and flagellar assembly regulatory genes (23). Neither isolate from the Bacteroidota (OXR-11 and OXR-203) shows evidence of genes related to the flagellar filament or motor complex. Bacteroidota uses gliding motility propelled by gliding proteins and adhesins expelled by a type IX secretion system (24, 25). Both *R. spongicola* OXR-11 and *Christiangramia* sp. OXR-203 carry gliding protein genes *gld* and *spr*, with *por* genes coding for the type IV secretion system (T9SS) (Table S4).

### Nutrient metabolism

KEGG pathway prediction revealed the Roseobacteraceae family (OXR-9, OXR-159, OXR-199, and OXR-209) encoded the genes necessary for complete thiosulfate oxidation (Table S6). These genes include *soxXYZABCD* coding for the multienzyme periplasmic proteins SoxAX, SoxYZ, SoxB, and SoxCD. Meanwhile, the energy-producing anaerobic dissimilatory nitrate reduction pathway was present in two isolates, *G. subterraneus* OXR-76 and *N. rhodophyticola* OXR-85, encoding for nitrate reductase genes *narGHI* and nitrite reductase genes *nirBD* (Table S7). *P. marinus* OXR-96, *Thalassobaculum* sp. OXR-137, *Sulfitobacter* sp. OXR-159, *S. pontiacus* OXR-199, and *L. variabilis* OXR-209 are missing one defined nitrate reductase KEGG block to complete the dissimilatory nitrate reduction pathway.

### Carbon fixation

Analysis of genomes with KEGG recovered portions of carbon fixation pathways including the Calvin-Benson-Bassham cycle, the reductive tricarboxylic acid (rTCA) cycle, the Wood-Ljungdahl pathway, the dicarboxylate-hydroxybutyrate cycle, and the 3-hydroxypropionate bi-cycle (Table S8). The majority of these genes were found on the primary chromosome. However, an acetyl-CoA C-acetyltransferase (K00626) which can be involved in the dicarboxylate-hydroxybutyrate cycle was also found on a number of plasmids from OXR-159, OXR-199, OXR-209, OXR-85, and OXR-9 (Table S8). Plasmids also contained aspects of the Calvin-Benson-Bassham cycle (K01624, K00134, and K00615), and 3-Hydroxypropionate bi-cycle (K02160 and K09709), although these, along with the former plasmid-bound genes, were likely involved in other metabolic processes.

### Secondary metabolism

**Fig 2:**
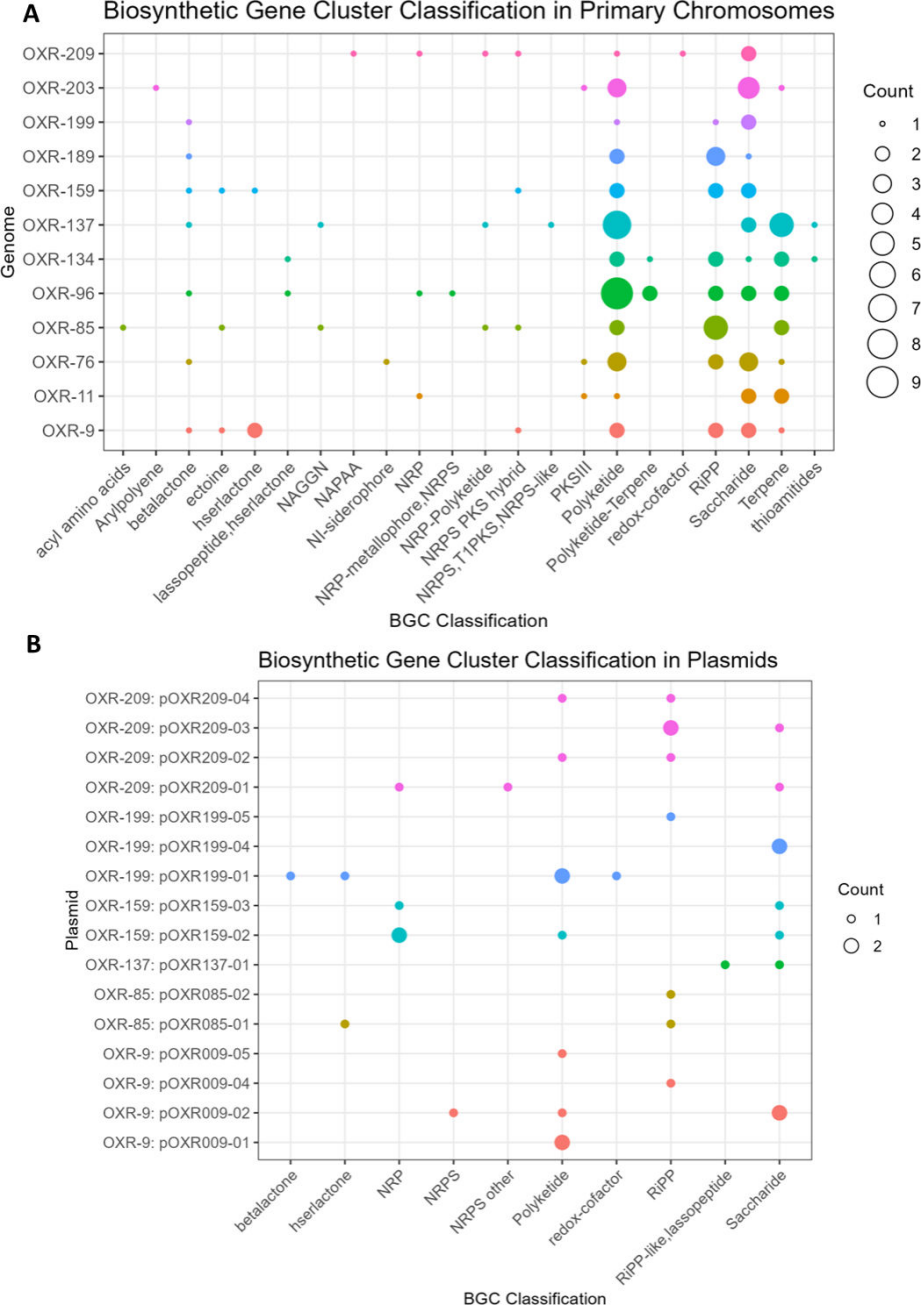
The biosynthetic gene cluster classification predicted to be in the primary chromosomes (**A**) and associated plasmids (**B**). Size of circles represents the number of BGCs with associated classification.

The combination of antiSMASH and DeepBGC predicted 359 BGCs (Table S9). However, 247 of the DeepBGC predictions were classified as unknown and not related to any cluster identified in the antiSMASH database. Among the 359 predicted biosynthetic gene clusters (BGCs), DeepBGC determined 93 to have a single gene (Table S9). Polyketides were the most abundant classification, followed by saccharides, RiPPs (ribosomally synthesized and post-translationally modified peptides), and terpenes in both the primary chromosome and plasmids (Fig. 2). The primary chromosomes of *S. faviae* OXR-9, *N. rhodopyticola* OXR-85 and *Sulfitobacter* sp. OXR-159 showed BGCs with 100% similarity to ectoine synthesis, a compatible solute and osmoprotectant (26) (Fig. 2A). *B. marina* OXR-134 and *Thalassobaculum* sp. OXR-137 also had BGCs with 100% similarity to the terpene zeaxanthin and another carotenoid, respectively (Fig. 2A). Two of the Roseobacteraceae, *S. pontiacus* OXR-199 and *L. variabilis* OXR-209, had more total predicted BGCs in their plasmids, including homoserine lactone, redox cofactors, RiPPs, and additional polyketides (Fig. 2B). The most common cluster in the plasmids was sphingan polysaccharide; found in three plasmids of the *Sulfitobacter* isolates, OXR-9 and OXR-159, with less than 15% similarity (Fig. S1B; Table S9).

**Fig 3:**
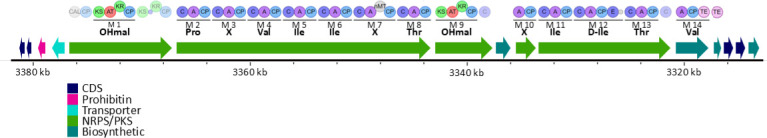
Genetic organization of the novel NRPS/PKS in *Thalassobaculum* sp. OXR-137. The top illustrates each of the functional modules used to incorporate the indicated amino acid. Modules included are co-enzyme A ligase domain (CAL), carrier protein (CP), ketosynthase (KS), acyltransferase (AT), ketoreductase (KR), condensation (C), adenylation (A), nitrogen methyltransferase (nMT), epimerase (E), and thioesterase (TE). Faded modules represent an incomplete module or outside modules. Amino acids incorporated are provided underneath, where X is an unknown amino acid and OHmal is hydroxymalonyl-CoA. Orientation of the genome is reversed for ease of reading.

Within *Thalassobaculum* sp. OXR-137 GI13, a putatively novel 95 kb NRPS/PKS cluster was found adjacent to a cyclic peptide export ABC transporter followed by a prohibitin family protein (Fig. 3). AntiSMASH analysis revealed that the closest known biosynthetic gene cluster in the database, tubulysin A, shared only 6% similarity, suggesting this cluster represents a distinct and potentially novel biosynthetic pathway (Fig. S1A; Table S9). The cluster includes 12 complete NRPS modules, with 9 predicted amino acids and 3 unpredicted, as well as 3 PKS modules. It features one missing acyltransferase (AT) domain and two thioesterase (TE) domains across seven ORFs. The NRPS/PKS ORFs are transcribed bidirectionally with the ABC transporter and prohibitin gene. The condensation (C) and ketosynthase (KS) domains in this cluster show less than 80% identity to other genes in the NCBI nr database, with the top hits belonging to *Niseae* sp. and other isolates from the Thalassobaculaceae family but none of the other *Thalassobaculum* genomes in NCBI.

### Transcriptional activity within the vent plume

**Fig 4:**
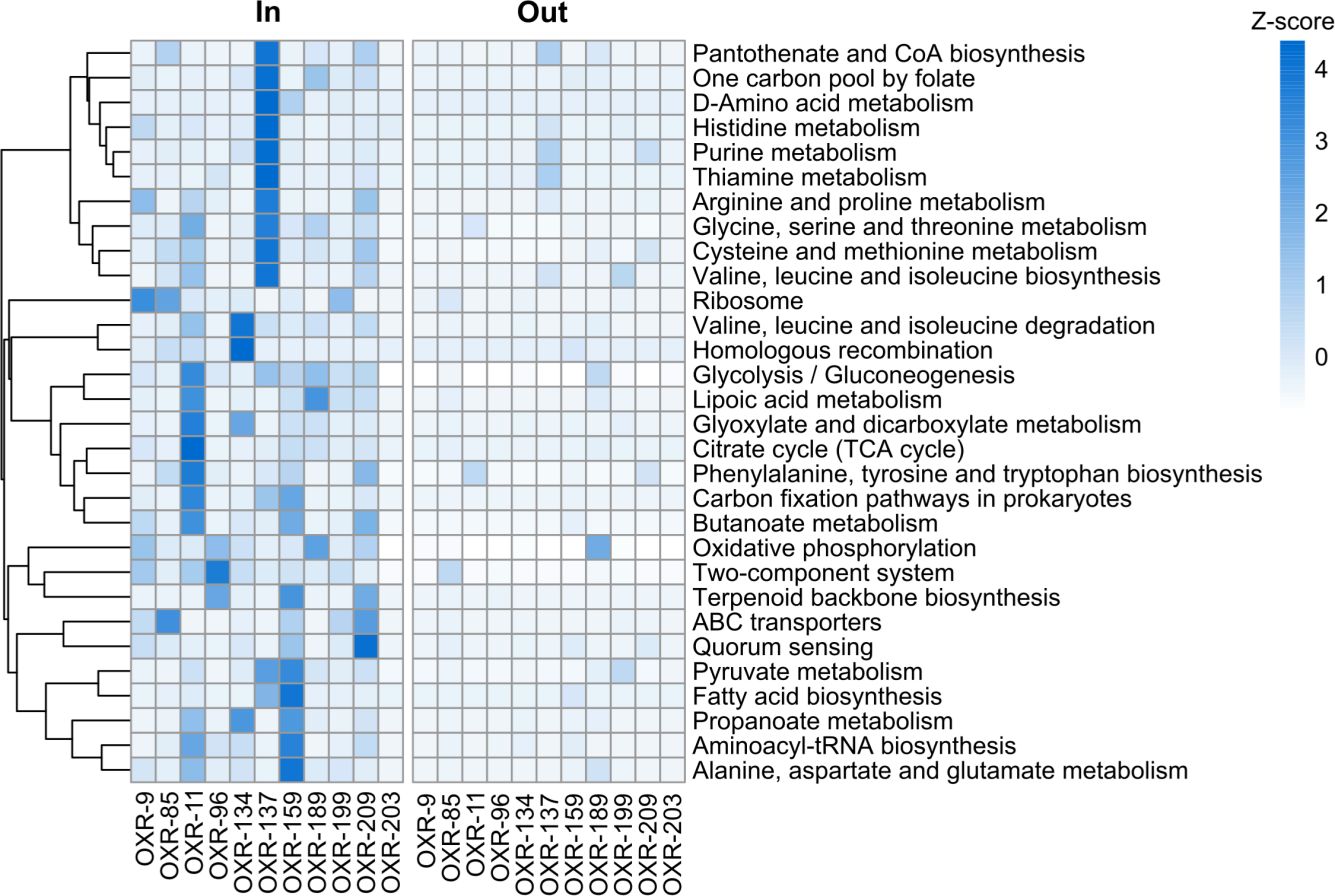
Heatmap of KEGG pathway-level responses displaying the row-wise *Z*-scores of TPM normalized read counts binned (sum of all counts) by KEGG pathway for bacterial isolates within the hydrothermal plume relative to without.

To assess the *in situ* activity and ecological relevance of the 12 bacterial isolates within the HVP, we aligned metatranscriptomic reads from 17 samples collected along a transect within the vent plume environment (15) to the sequenced genomes. Transcriptional responses were observed for all bacterial isolates across all 17 samples (Fig. S2). However, there was no significant difference in expression between transcripts found in the plume versus those found outside of the plume (*P*adj >0.05, Table S4). Transcriptional activity was mainly observed within samples located in the vent plume, although highly variable, with two isolates (OXR-85 and OXR-189) displaying activity at locations outside the plume (Fig. 4).

### GIs of four novel isolates

Four out of 12 isolates were identified as putatively novel based on dDDH values compared to their closest type-strain. To further validate this, average nucleotide identity (ANI) was calculated against the respective type-strain and closest 16S rRNA gene relatives with available genomes in GenBank (Table 2). Comparative genomics was then conducted to explore potential evolutionary differences, focusing on the identification of GIs containing unique genes. GIs, defined as regions with 10 or more non-syntenic genes separated by fewer than 10 genes, are of particular interest due to their role in acquiring potentially adaptive traits through HGT events (8). These traits can include integrative conjugative elements (ICEs), transposons, prophage integrations, and other mobile genetic elements (MGEs), or may result from past gene deletion events (27). Results for each putatively novel isolate are detailed below.

### *Thalassobaculum* sp. OXR-137 GIs

**Fig 5:**
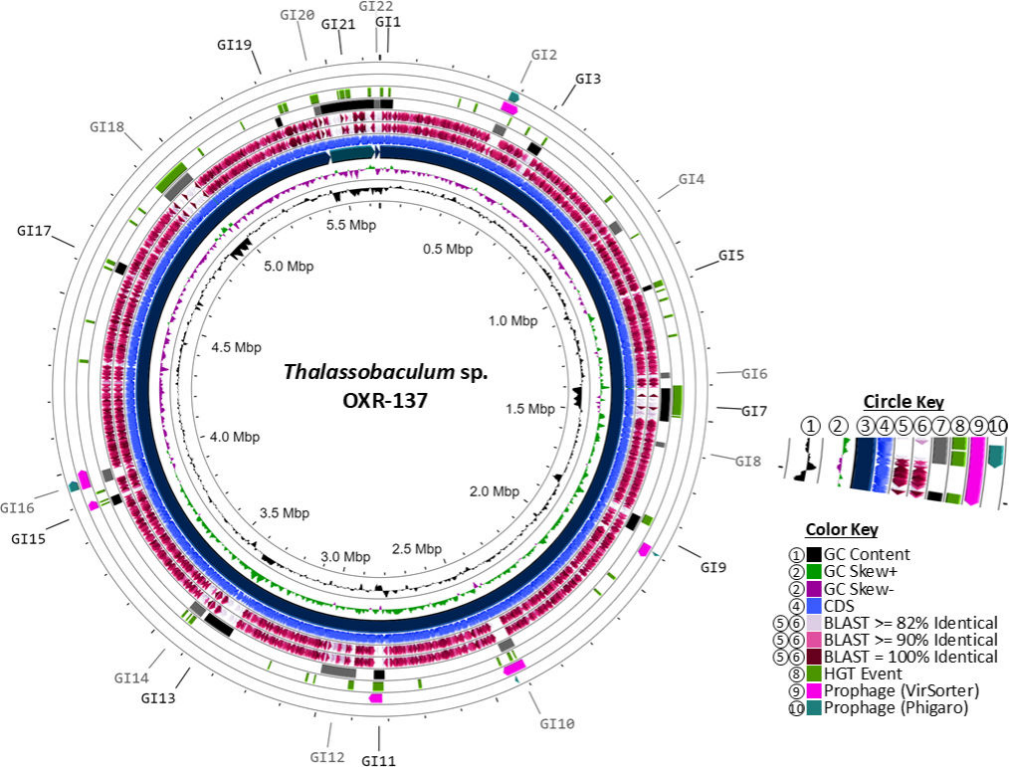
Circular representations of novel isolate *Thalassobaculum* sp. OXR-137, including plasmids as smaller segments. The circle key (1–10) corresponds to concentric circles starting closest to the center with (1), radiating outward. (1) GC content; (2) GC skew; (3) genomic contigs, sorted by size; (4) CDS; (5) BLASTx percent ID of type-strain; (6) BLASTx percent ID of 16S relative; (7) genomic islands; (8) Alien Hunter predicted HGT events; (9) VirSorter prophage prediction; and (10) Phigaro prophage prediction. The color key indicates the colors of the feature found in the circles.

*Thalassobaculum* sp. OXR-137 shows an ANI of 89.25% (33.2% dDDH) with its type-strain *T. litoreum* DSM 18839, and ANI and dDDH values of 89.36% and 33.4%, respectively, with its closest relative based on 16S rRNA gene sequence, *T. salexigens* DSM 19539 (Table 2). The primary chromosome of *Thalassobaculum* sp. OXR-137 contains 19 GIs, encompassing 948 genes, 291 of which are hypothetical (Table S10). Notably, GI1 and GI20, positioned at the extremities of the linear chromosomal representation, form a contiguous GI and will be referred to as GI20-1 (Fig. 5). The analysis identified three GIs (GI20-1, GI7, and GI18) that possess putative ICEs (Tables S10 and S11) and five predicted to be associated with phage (GI2, GI10, GI11, GI15, and GI16).

GI20-1 has copper resistance proteins A and B, cupredoxin, and arsenic resistance proteins ArsC, ArsJ, and ArsR (Table S10). GI7 is a 104 kb region with 102 predicted genes, encompassing a type IV toxin-antitoxin system, tripartite ATP-independent periplasmic transporter system proteins, chaperonins GroES and GroEL, and a thermonuclease protein. GI18, also a 104 kb region, contains 105 predicted genes, including components of the BREX phage defense system. GI7 and GI18 may possess ICEs, as evidenced by the presence of type IV secretion system proteins, recombinases, integrases, conjugation proteins, and accessory genes (28). Additionally, GI12 is a non-ICE GI potentially facilitating adaptation in hydrothermal vent systems, encoding nickel and iron ABC transport systems. GI13 contains a novel NRPS/PKS system but is not predicted to be associated with an HGT event (Tables S10 and S11).

The plasmid GIs, GI21 and GI22, located within plasmids pOXR137-01 and pOXR137-02, respectively, appear to be conjugative, featuring type IV secretion systems. GI21 is a 168 kb region encoding nitrate reductases, nitric oxide remediation enzymes, toxin-antitoxin (TA) systems, lassopeptides, ATP-independent transporters, and resistance mechanisms for heavy metals such as copper, cobalt, zinc, cadmium, mercury, and nickel. In contrast, GI22 is an 18 kb region with 25 predicted genes primarily associated with mercury resistance.

### *Sulfitobacter* sp. OXR-159 GIs

**Fig 6:**
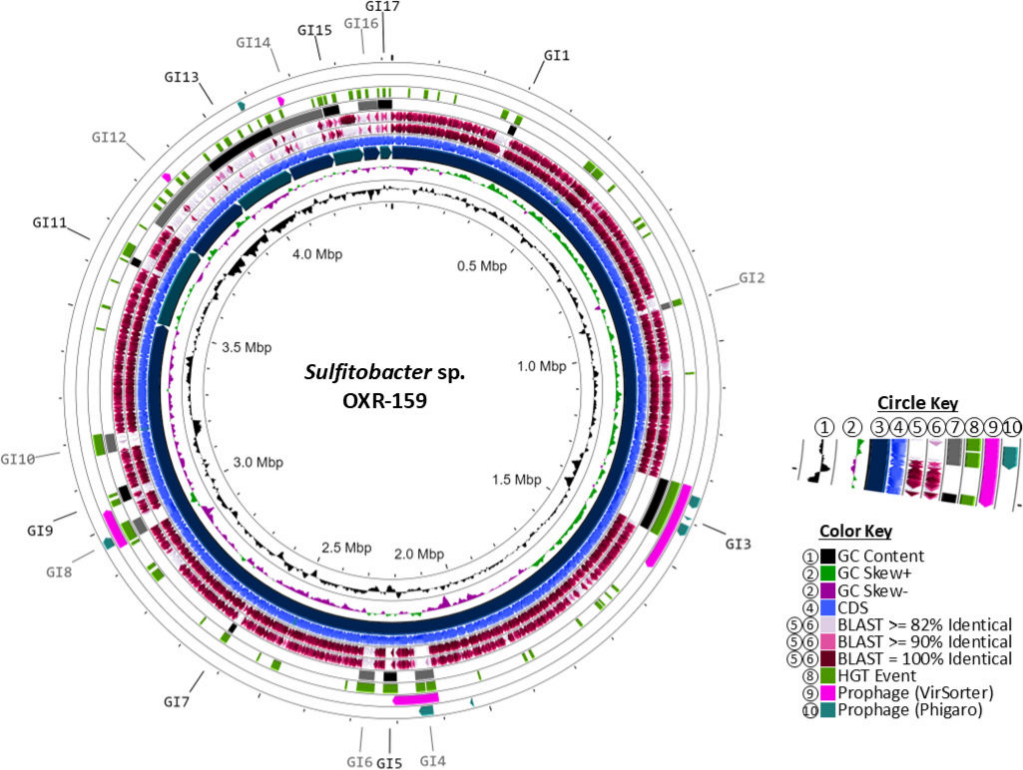
Circular representations of novel isolate *Sulfitobacter* sp. OXR-159, including plasmids as smaller segments. The circle key (1–10) corresponds to concentric circles starting closest to the center with (1), radiating outward. (1) GC content; (2) GC skew; (3) genomic contigs, sorted by size; (4) CDS; (5) BLASTx percent ID of type-strain; (6) BLASTx percent ID of 16S relative; (7) genomic islands; (8) Alien Hunter predicted HGT events; (9) VirSorter prophage prediction; and (10) Phigaro prophage prediction. The color key indicates the colors of the feature found in the circles.

*Sulfitobacter* sp. OXR-159 exhibits an ANI of 96.3% (67.6% dDDH) relative to its type-strain *S. indolifex* SAORIC-263 and ANI and dDDH values of 90.1% and 36.3%, respectively, compared to *S. dubious* CI.11.F.A3 (Table 2). The accepted ANI threshold for a new species is 95–96% (29, 30), indicating a discrepancy between dDDH and ANI novel species predictions for *Sulfitobacter* sp. OXR-159. The primary chromosome of *Sulfitobacter* sp. OXR-159 contains 10 GIs (GI1–10) comprising 626 genes, 208 of which are hypothetical (Table S10). Additionally, the seven plasmids each harbor a GI (GI11–17). Notably, the plasmids are themselves GIs, excluding pOXR159-01 (GI11) and pOXR159-05 (GI15), with a total of 763 genes and 241 hypothetical proteins (Fig. 6; Table S10). Among these, three GIs may represent ICEs, and seven appear to be prophage-related as predicted by Phigaro and Virsorter2, with one also featuring a BREX phage protection system (Tables S10 and S11). Evidence of HGT is found across all islands. Key findings include GI9, a 37.4 kb region with 42 genes that encompasses the QseB and QseC phosphorelay system, potentially enhancing the production of the vitamin B12 importer BtuD (31), as well as drug resistance proteins, multidrug efflux systems, and an antifreeze protein. GI11, an 18.8 kb island on plasmid pOXR159-01, contains genes involved in nitrate and nitrite uptake and nitrite reductase proteins. GI12, a 191 kb region on plasmid pOXR159-02, features 232 genes, including TA systems, ABC transporters for nickel, peptides, and amino acids, ATP-independent transport systems, and a type I secretion system. GI13, a 175 kb island on plasmid pOXR159-03, includes 210 genes, with peptide and nickel ABC transporters, a TA system, cytochrome C, and copper resistance proteins. Finally, GI15, a 39.2 kb region on plasmid pOXR159-05, has 46 genes related to a TA system, a vitamin B12 import system, and transport systems for iron, cadmium, cobalt, and zinc. In addition, these GIs encode two TA systems and transport systems for iron, cadmium, cobalt, and zinc, underscoring their roles in stress response and metal resistance.

### *Idiomarina* sp. OXR-189 GIs

**Fig 7:**
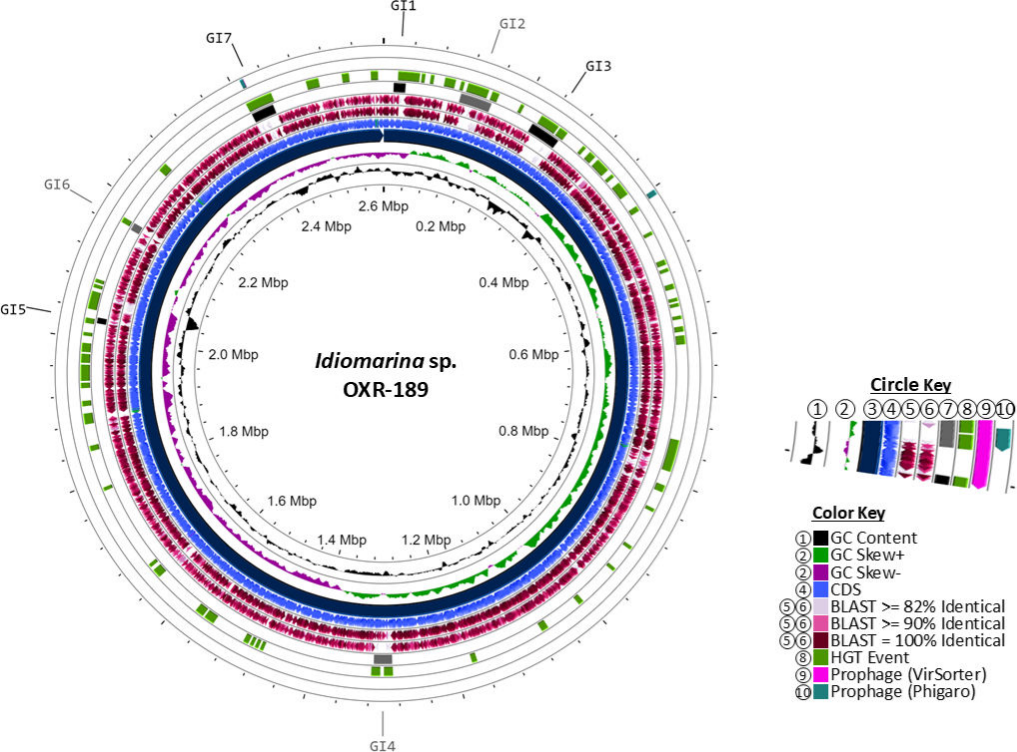
Circular representations of novel isolate *Idiomarina* sp. OXR-189. The circle key (1–10) corresponds to concentric circles starting closest to the center with (1), radiating outward. (1) GC content; (2) GC skew; (3) genomic contigs, sorted by size; (4) CDS; (5) BLASTx percent ID of type-strain; (6) BLASTx percent ID of 16S relative; (7) genomic islands; (8) Alien Hunter predicted HGT events; (9) VirSorter prophage prediction; and (10) phigaro prophage prediction. The color key indicates the colors of the feature found in the circles.

*Idiomarina sp*. OXR-189 displays an ANI of 96.7% (68.8% dDDH) relative to its type-strain *I. zobellii* KMM 231. It has ANI and dDDH values of 87.8% and 31%, respectively, compared to *Idiomarina* sp. X4 (Table 2). The chromosome of *Idiomarina* sp. OXR-189 contains seven GIs (GI1–7) encompassing 204 genes; 66 are hypothetical. HGT events were identified in all GIs, although GI1 and GI2 did not show mobile genes as predicted by MobileOG-db (Table S10). While VirSorter2 did not predict any prophages, Phigaro indicated prophage presence in GI7 (Fig. 7; Tables S12 and S13). Manual inspection of GI7 revealed it contains only an inovirus-type Gp2 protein and a prophage regulatory protein AlpA (Table S10). None of the GIs harbor type IV secretion systems or other conjugative elements, suggesting the absence of ICEs. GI1 is a 17.1 kb region with 16 genes, including a vitamin B12 transporter BtuB protein and a protein involved in cadmium, cobalt, and zinc efflux. GI2 spans 45.5 kb with 44 genes, including methyl-accepting chemotaxis proteins and several peptidase/protease variants. GI3, a 43.9 kb region with 49 genes, includes a TA system and the CsrA global regulator. GI4 is a 263 kb region with 30 genes that, while not predicted as a prophage, codes for a prophage integrase, a transcriptional regulator, the inovirus Gp2 protein, additional methyl-accepting chemotaxis proteins, another CsrA regulator, and three subunits for a type I restriction-modification system. GI5 and GI6, each less than 11 kb with fewer than 20 genes, include multiple transposases and potential insertion sequences; GI5 also features a cation transporter and heavy metal transcriptional regulators. GI7, a 32.9 kb region with 37 genes, encompasses a heavy metal efflux system along with the prophage-related genes.

### *Christiangramia* sp. OXR-203 GIs

**Fig 8:**
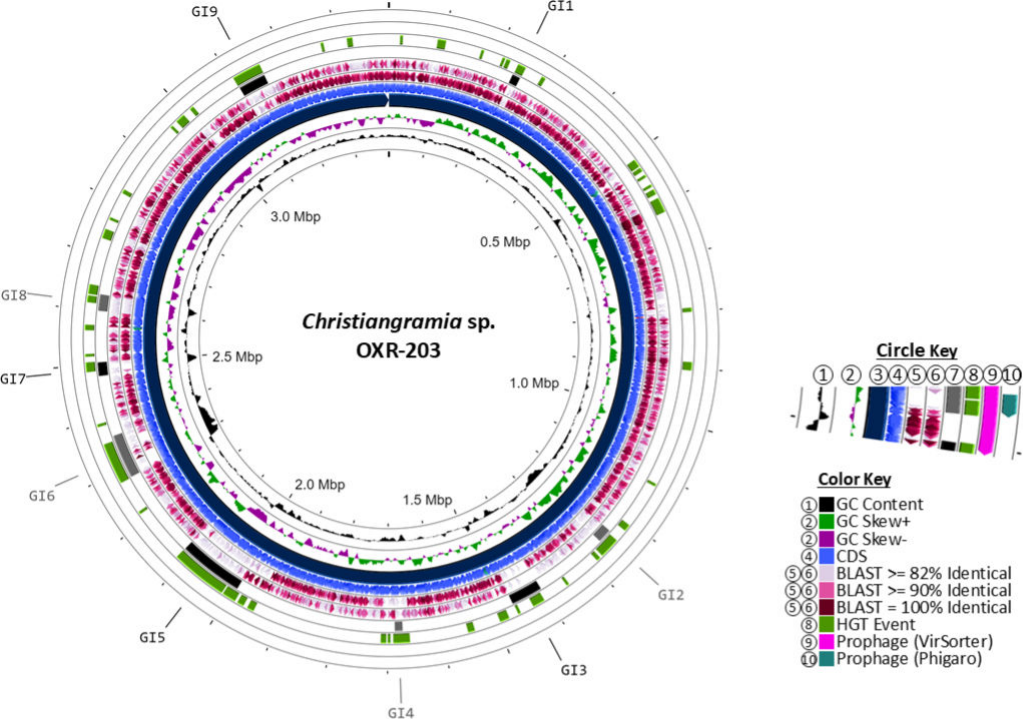
Circular representations of novel isolate *Christiangramia* sp. OXR-203. The circle key (1–10) corresponds to concentric circles starting closest to the center with (1), radiating outward. (1) GC content; (2) GC skew; (3) genomic contigs, sorted by size; (4) CDS; (5) BLASTx percent ID of type-strain; (6) BLASTx percent ID of 16S relative; (7) genomic islands; (8) Alien Hunter predicted HGT events; (9) VirSorter prophage prediction; and (10) Phigaro prophage prediction. The color key indicates the colors of the feature found in the circles.

*Christiangramia* sp. OXR-203 displays an ANI of 85.5% (28.1% dDDH) relative to its type-strain *C. portivictoriae* DSM 23547. Additionally, it has ANI and dDDH values of 80.1% and 19.4%, respectively, compared to *C. echinicola* MAR_2010_102 (Table 2). The genome of *Christiangramia* sp. OXR-203 comprises nine GIs, designated GI1-GI9, encompassing a total of 504 genes, of which 139 are hypothetical (Fig. 8; Table S10).

Alien Hunter (32) predicted each genomic island contained an HGT event. MobileOG-db (33) analysis indicated the presence of MGEs in GI5, GI6, and GI9, specifically single-stranded DNA binding proteins, a DNA methylase, and an endoribonuclease (Table S10). Notably, neither Phigaro (34) nor VirSorter2 (35) predicted the presence of prophage-related gene within the genome. Detailed genomic island analyses revealed specific functional attributes (Table S10): GI1 is a 15.2 kb region containing 16 genes, including a multidrug efflux transporter, the outer membrane protein TolC, and a recombinase. GI2 spans 26.3 kb with 28 genes involved in the biosynthesis and modification of nucleotide sugars and related compounds. GI3 is a 59 kb region comprising 49 genes, including the stress response sigma factor (σ^54^), the global transcription regulator σ^70^, and ABC transporter proteins. GI4 encompasses 14.4 kb with 24 genes, featuring several transposases, an insertion sequence element protein TnpB, and enzymes involved in the cleavage and degradation of aromatic compounds. GI5 is a 122 kb region containing 162 genes related to multidrug and heavy metal efflux systems, including transport proteins for cadmium, cobalt, copper, lead, mercury, silver, and zinc. GI6 spans 82.6 kb with 115 genes, including an erythromycin esterase and three different TA systems. GI7 is a 25.4 kb region with 22 genes, including an Eco571 restriction-modification methylase. GI8 is a 30.7 kb region containing 36 genes, lacking transposases but including a gene involved in sialic acid synthesis. GI9 is a 51.2 kb region with 52 genes, potentially functioning as an ICE by coding for a type IV conjugative system. It also includes a heavy metal efflux system, superoxide dismutase, and a transporter protein for the siderophore enterobactin.

## DISCUSSION

The analysis of 12 bacterial genomes cultured from the Moytirra HVP highlights the role these environments play in hosting diverse and potentially novel microbial species, emphasizing the distinct role of hydrothermal vents as reservoirs of microbial diversity and genomic innovation. By complementing culture-independent data with culture-derived findings, we were able to confirm the presence and activity of the sequenced genomes within the plume.

### Novel species and comparative genomics

The discovery of putatively novel isolates from the genera *Thalassobaculum*, *Sulfitobacter*, *Idiomarina*, and *Christiangramia,* based on dDDH and ANI, reinforces the concept that HVPs are hotspots for biodiversity and genomic innovation. In our survey, *Idiomarina* sp. OXR-189 was initially classified as a putatively novel species based on dDDH values of 68.8% when compared to its type-strain *I. zobellii* KMM231. However, ANI evaluation against the type-strain showed 96.7% identity, suggesting they are part of the same species. *Idiomarina* sp. OXR-189 has the smallest genome and the fewest functional assignments related to carbohydrate metabolism out of the 12 genomes studied. Members of the *Idiomarina* genus are frequently found as free-living bacteria in the deep-sea or high-saline marine environments with reduced genomes compared to other Alteromonadales (36–38). It has recently been shown that some *Idiomarina* exclusively use amino acids as a preferred carbon source (39), indicating they have undergone trophic specialization by eliminating genes involved in carbohydrate metabolism (38), at least partially explaining the reduced genome size. The presence of *Idiomarina* sp. OXR-189 within the HVP suggests that it may perform a specialized function to recycle deep-sea peptides into simpler forms for utilization by other community members.

*Sulfitobacter sp*. OXR-159 was also initially identified as a novel species based on dDDH values. However, an ANI value of 96.3% compared to *S. indolifex* SAORIC-263 suggests it may be the same species. This was an unexpected finding due to nearly 25% of the genome being associated with GIs that are associated with predicted prophage and/or HGT events, highlighting limitations of genome-based species delineation. Among the 17 GIs found in this isolate, 7 were found on plasmids that have low sequence identity to the compared genomes. Given that these plasmids were not found in the type-strain, it may identify them as species distinguishing features or that they were acquired through a conjugation event where *Sulfitobacter* sp. OXR-159 was the recipient of the horizontally transferred replicons. Missing conjugation machinery in the largest of the plasmids (pOXR159-01, pOXR159-02, pOXR159-03, and pOXR159-05) implies that these may have become stable components of the isolate’s genome by providing advantages such as phage defense, heavy metal resistance, and genes for nitrite uptake and reduction. In contrast, the smaller plasmids with conjugative potential may be more promiscuous, moving from cell to cell, enhancing the host bacterium’s energy economy by carrying transporters for branched-chain amino acids, tricarboxylates, and ATP-independent transporters that ultimately lower its dependency on host energy stores to support its replication.

The comparison of *Christiangramia* sp. OXR-203 against two closely related species, *C. portivictoriae* and *C. echinicola*, supported its classification as a novel species, despite the 99% identity of the 16S rRNA sequences to both. Nine GIs were identified and are not of viral origin but potentially created through HGT events such as ICE integration. The lack of phage within the genome may be due to protection conferred by TA and restriction modification systems, like those encoded in the GIs. The GIs also coded for multidrug efflux proteins and an erythromycin esterase, likely conferring drug resistance in the context of their proximity to other bacteria when attached to marine particulate matter (40, 41). Future investigation into the GIs within the bacteria of similar communities will aid in determining the prevalence and potential origin of such defense mechanisms.

The largest genome sequenced in this study belonged to the putatively novel isolate *Thalassobaculum* sp. OXR-137. ANI and dDDH metrics compared against type-strain *T. litoreum* DSM 18839 supported the classification of *Thalassobaculum* sp. OXR-137 as a novel species. This isolate exhibited distinct functional patterns compared to the other Pseudomonadota isolates and was the only isolate to show evidence of bacteriochlorophyll genes (*bch*; Table S14); a common characteristic among the Rhodospirillales order where they are able to perform anoxygenic photosynthesis (42). The presence of *bch* may allow *Thalassobaculum* sp. OXR-137 to harness geothermal light-driven photosynthesis for additional energy (43, 44). Unlike the other isolates, *Thalassobaculum* sp. OXR-137 harbors multiple copies of the flagellin protein gene (*flaA*) and the greatest count of chemotaxis-related genes on the primary chromosome, suggesting an adaptation to a free-living pelagic lifestyle by enabling the ability to sense and respond to chemical fluctuations in the surrounding environment. The ability to respond to deleterious conditions may also be reflected in its 21 GIs, including the two potentially mobilizable plasmids. Among the GIs are putatively novel NRPS/PKS and various phage defense mechanisms, which spread across the primary chromosome and the plasmids. There were also heavy metal resistance genes found across the GIs but on different genomic replicons, including copper resistance genes present on the primary chromosome and the plasmids, arsenic resistance found on the primary chromosome, and mercury resistance genes isolated on the plasmids. Both copper and arsenic resistance evolved early in Earth’s history; where copper is toxic to cells but evolved to be a co-factor in many enzymes (45), and arsenic toxicity is mitigated by the evolution of a single, horizontally transferred, universal arsenate reductase (*arsC*) ancestor (46). A previous study found that greater than 70% of isolated strains from deep-sea hydrothermal vents are resistant to mercury toxicity, and it was proposed that mercury-reducing bacteria may be playing an ecological role in detoxifying mercury to allow the deep-sea vent fauna to thrive (47). If the aforementioned plasmids containing heavy metal resistance genes are actively transferred within the community, as suggested for arsenic resistance (46), their distribution among the population may represent an important functional role *Thalassobaculum* sp. OXR-137 may play in the local environment.

In addition to detecting rRNA operons on all primary chromosomes, rRNA operons were also detected on plasmids predicted in *S. pontiacus* OXR-199 and *L. variabilis* OXR-209. Although these operons are considered defining features of “chromosomes” (48, 49), they have been observed on plasmids in other bacteria (50, 51) as well as existing on the plasmid alone (52) which is thought to be stably maintained for long evolutionary periods (53). The presence of additional rRNA on plasmids may provide a selective advantage by providing more rapid adaptation to environmental shifts, as seen in *Escherichia coli*, which contains seven copies of rRNA (54). Further, additional rRNA on plasmids may help facilitate the exchange of genetic material between bacteria (55) potentially conferring more rapid adaptation as well. This could be particularly relevant in hydrothermal vent systems which have narrow chemical and physical niches (56).

### Functional traits

Functional inquiries into the metabolic processes of the 12 genomes revealed a propensity for chemosynthetic trophism. All members of the Roseobacteraceae family were found to possess a complete thiosulfate oxidation pathway (*soxXYZABCD*), highlighting their ability to oxidize sulfur compounds (such as thiosulfates, sulfites, and sulfates) for energy production (57, 58). Known for their adaptability, Roseobacteraceae can support their growth while fixing CO_2_ by performing thiosulfate oxidation under both aerobic and anaerobic conditions (59, 60). Previous metatranscriptomic analyses of the Moytirra HVP have demonstrated that *sox* genes, apart from *soxX* and *soxZ*, are upregulated (15), suggesting these genes play an important metabolic role for microbial communities within the vent.

Additionally, two isolates were found to have complete dissimilatory nitrate reduction pathways, *N. rhodophyticola* OXR-85 and *G. subterraneus* OXR-76, suggesting alternative energy metabolisms may be important within this ecosystem as observed in prior vent systems (61). However, within the Moytirra vent plume, Polinski et al. (15) observed that community-level *narG* and *narH* were significantly downregulated (15), indicating that anaerobic dissimilatory nitrate reduction was likely not occurring. In addition, when these metatranscriptomes were aligned to our genomes, no genes were significantly expressed, suggestive of low functional activity in these isolates *in situ*. As such, although these isolates are capable of nitrate reduction, it does not appear to be actively used within the Moytirra plume.

Across the 12 genomes, all isolates were found to possess genes involved in multiple carbon fixation pathways common to vent microbial organisms. Carbon fixation pathways tend to be distributed by geochemistry and temperature (62). For instance, the rTCA cycle is typically found in warmer waters (20–90°C), where oxygen levels are sufficiently low (16) while the energetically costly oxygen-tolerant Calvin-Benson-Bassham cycle is found at temperatures lower than 20°C. At much warmer temperatures (>90°C), microorganisms performing sulfate reduction tend to rely on the Wood-Ljungdahl pathway and the dicarboxylate–4-hydroxybutyrate pathway (62). As such, the presence of one or more carbon fixation pathways within the genomes may indicate innate environmental plasticity necessary for survival and/or persistence in these extreme environments, such as observed in vent endosymbionts which can switch between several carbon fixation pathways (63).

*G. subterraneus* OXR-76, the only Bacillota and thermophile among the isolates, was found within the plume at 3.27°C but could not be grown at 20°C. Few transcripts mapped to its genome, and thus we do not expect it was functionally active at the time of sampling. It is possible that *G. subterraneus* OXR-76 was carried into the plume from warmer areas surrounding the vent where it performed dissimilatory nitrate reduction. Although *G. subterraneus* KCTC 3922T has been shown to have potential for denitrification (64), *G. subterraneus* OXR-76 was missing one KEGG ortholog necessary for a complete denitrification pathway.

Each of the 12 isolates’ genomes show evidence for motility, either through flagellar ambulation or gliding mechanisms. However, differences in the regulatory expression mechanisms can exist among the different bacterial groups (65). For instance, the Roseobacteraceae were found to lack the stress response and flagellar regulatory gene *rpoN* (22) while maintaining the primary housekeeping sigma factor gene *rpoD*. The Bacillota does not carry the house-keeping *rpoD* because its house-keeping and flagellar assembly regulation is controlled by the alternative sigma factors coded by *sigA* and *sigD* (23).

The flagellar assembly genes in *S. pontiacus* OXR-199 are located on a previously undescribed plasmid rather than the primary chromosome. Similar flagellar organization has been observed in polar psychrophilic Roseobacteraceae (66) suggesting that motility itself may be a mobile element in some bacterial communities, conferring adaptive advantages during dispersal in pelagic environments. Conversely, *R. spongicola* OXR-11 and *Christiangramia* sp. OXR-203 lack flagellar assembly genes but carry genes involved in gliding motility typically found in the Bacteroidota phylum (67). Gliding is associated with type IX secretion systems that release carbohydrate-active enzymes and adhesins to propel the cell forward (68). As the third most abundant group of bacteria in the ocean, Bacteroidota are often associated with surfaces of particles and algal cells, and in addition to providing gliding motility, the secretion of enzymes allows them to break down high-molecular weight compounds derived from these substrates (40). The relative enrichment of *R. spongicola* OXR-11 transcripts related to carbohydrate metabolism may reflect the complex substrates available within the plume. This is exemplified by the relatively large proportion of differentially expressed genes related to carbohydrate metabolism found within the plume (15), though it is unclear if these are prokaryotic or eukaryotic in origin. In summary, these findings suggest that the bacteria cultured from the Moytirra HVP represent isolates with potentially complex metabolic roles within the vent ecosystem.

### Dispersal and MGEs

Detailed inspection of the novel isolates showed a variety of MGEs such as transposons, ICEs, and the integration of prophage into the genomes. Nearly all the GIs are associated with an HGT event, exhibiting the crucial role HGT plays in facilitating the acquisition and dissemination of genes related to the adaptation of the local environment. The large-scale capability of being able to horizontally transfer genetic information between populations can lead to, for instance, the dissemination of monophyletic heavy metal resistance across the three domains of life (46). At a smaller scale, the GIs may be reflective of a populations’ genetic microdiversity in which MGEs target the GIs and induce strain-specific or population-level adaptations (8). Similarly, the evidence of HGT events linked to secondary metabolite production in several isolates suggests that HGT plays a key role in spreading functional traits that confer advantages in the fluctuating and chemically diverse conditions of HVPs (69). This supports the notion of HVPs as dynamic reservoirs of genomic innovation, where microbial communities are shaped by both environmental pressures and the exchange of genetic material (56). However, future research into the GIs, their functions, and products will provide greater understanding of the mobility, prevalence, and evolution across HVPs and vent field environments.

### Secondary metabolism

AntiSMASH and DeepBGC predicted BGCs in each genome. Although machine learning tools such as DeepBGC are rapidly advancing, some limitations persist. For instance, DeepBGC predicted many single-gene clusters or clusters without clear biosynthetic pathways. However, it was able to predict the same BGCs as antiSMASH. The discovery of a novel NRPS/PKS gene cluster in one genome underscores the biotechnological potential of microbes from extreme environments, emphasizing hydrothermal vents as promising sources for natural product discovery like pigments, antibiotics, and anticancer agents (70). *Thalassobaculum* sp. OXR-137, with the highest number of BGCs (*n* = 49), features a putative novel NRPS/PKS cluster in genomic island GI13, located upstream of a potentially novel prohibitin gene. Prohibitins, which act as scaffolding proteins vital for mitochondrial function (71, 72), may interact with the NRPS/PKS cluster. The extensive metabolic capabilities and the presence of this novel gene cluster in *Thalassobaculum* sp. OXR-137 suggest that further research could reveal its environmental role and biotechnological applications.

### Conclusion

This study cultured bacteria from the plume of a hydrothermal vent and sequenced select genomes to explore their function and architecture. Twelve isolates’ genomic repertoire were surveyed for their metabolic potential, which exhibited diverse capabilities and supplemental functions carried on plasmids. This study also identified four potential new species of bacteria, with one containing a putatively novel NRPS/PKS BGC, highlighting the genomic knowledge gap of microbial communities associated with deep-sea environments. These findings provide genome-specific insights into bacterial functions and adaptations in HVP environments, whether as transient residents from the surrounding water or permanent benthic community members expelled by vent emissions, emphasizing their crucial role in sustaining the chemosynthetic foundation of the plume’s ecosystem.

## MATERIALS AND METHODS

### Sample collection, culturing, and isolation

Samples were collected aboard the R/V OceanXplorer (OXR20210703) on 6 July 2021, from the HVP emanating from the Moytirra vent system (Lat 45.4833°N, Lon −27.8500°W) using Seabird 911 + CTD rosette with 12 L Niskin bottles as described in Polinski et al. 15. The plume sample used for bacterial isolation was collected at a depth of 2,488 m, with salinity 34.94 PSU and temperature 3.27°C. Collected plume seawater (1 L) was filtered across a 47 mm 0.2 µm pore size ME filter, then cryopreserved in 50% glycerol diluted in filter-sterilized seawater at −80°C or colder until revival.

For revival, filters were thawed on ice and then vortexed at maximum speed for 5 min to dislodge bacteria. The glycerol was diluted 1:10 in artificial sea water (ASW, 35 ppt salinity at 20°C). The undiluted and 1:10 diluted glycerol were then plated onto a marine medium according to Demko et al. 18 at varying concentrations (Table S1). Cultures grew for 7–28 days at 5°C, 20°C, and 60°C and were subcultured for isolation, prioritizing pigmented colonies and morphologies unique to the culture plate. Colonies were transferred to Difco Marine Broth until the culture was turbid to the eye (3–18 days). Isolates were then cryopreserved in 25% glycerol and frozen at −70°C. Separate aliquots of the isolates were frozen at −20°C for 16S rRNA gene sequencing.

### 16S rRNA gene identification

Frozen cell aliquots were thawed at room temperature, centrifuged at 10,000 × *g* for 5 min, and supernatant was decanted. A total of 10 µL of concentrated liquid culture was added to 100 µL of ultrapure water, and cells were lysed by heating to 95°C for 15 min, then placed on ice. 16S rRNA gene amplification was performed with forward primer v4_515F (73) and reverse primer v4_806R (74), modified to contain linking sequences for Illumina adapter addition, as described in Polinski et al. 15. PCR reactions contained 1X OneTaq HS master mix (New England Biolabs, Ipswich MA), 0.2 µM each forward and reverse primers, and 2 µL crude cell lysate in 25 µL. PCR conditions were as follows: initial denaturation at 94°C for 3 min, followed by 30 cycles of 94°C for 30 s, 60°C for 30 s, and 72°C for 30 s, with final extension at 72°C for 5 min. PCR product (5 µL) was then used in a second 20 µL reaction with 1× OneTaq HS master mix and 0.25 µM each Illumina adapters with unique dual indices for each sample. PCR reaction conditions were the same as the first PCR, but only seven cycles were needed for adapter and barcode addition. Products were pooled based on band intensity, excess primer and adapter were removed with a 1× PCRclean DX bead cleanup, the pooled library was quantified with NEBNext Library Quant for Illumina kit (New England Biolabs) sequenced on a 2 × 250 MiSeq run with v2 reagent kit (Illumina, Inc., San Diego, CA, USA).

Read trimming and ASV calling were performed with the DADA2 v1.30 R package (75), removing the first 10 bases from each read and the last 50 and 20 bases of the forward and reverse reads, respectively, allowing a maximum expected error rate of 2. Taxonomy was assigned with the Silva v138.1 database (76). To determine the presence of the cultured isolates within the Moytirra HVP plume community, ASVs were aligned against metabarcoding data of the Moytirra HVP (15) using BLASTn (77) with default parameters.

### DNA extraction for WGS

Twelve bacterial isolates were chosen for WGS based on their novelty relative to the SILVA v138 database, uniqueness of the 16S sequence among all isolates, and relevance to the marine environment (Table S2). Isolates were revived from glycerol stocks in Difco Marine Broth for 3–7 days, shaking at 160 rpm at 20°C (Thermo Scientific MaxQ 8000 incubator shaker), except for isolate OXR-76, which was revived at 60°C. The cells were pelleted, the supernatant was decanted, and pellets were stored at −70°C until DNA extraction. Purity was checked by streaking on marine agar plates.

DNA was extracted from thawed pellets using a modified CTAB extraction (78). Frozen 20 µL aliquots of each of the 12 isolates was thawed at room temperature, and 600 µL of CTAB solution (100 mM Tris, 1.4 M NaCl, 20 mM EDTA, and 2% CTAB) was added to each aliquot with 0.6 µL β-mercaptoethanol and vortexed to resuspend the pellet. A total of 15 µL of 20 mg mL^−1^ of proteinase K was then added and incubated at 65°C for 2.5 h, followed by an addition of 1.2 µL of 10 mg mL^−1^ of RNase and incubated at 37°C for 15 min. A total of 600 µL chloroform was added and mixed by inversion for 15 min. Tubes were spun for 10 minutes at 16,627 × *g* to separate the aqueous and organic layers. Isolated DNA was precipitated by storing at −20°C for 72 h then centrifuged at 4°C, 16,627 × *g*, for 20 min. The supernatant was decanted and washed with fresh 70% ethanol and spun again for 5 min. The supernatant was decanted again, allowed to dry, and resuspended in 50 µL of MilliQ water. The DNA was quantified using the dsDNA BR Qubit Quantification Kit (Life Technologies, Eugene, OR, USA). Samples OXR-96 and OXR-134 generated a viscous mass in the aqueous layer during the phase separation step. The viscous layer was transferred to a new tube and underwent a secondary CTAB extraction starting with dissolving the viscous portion in 600 µL CTAB solution overnight and mixing by inversion. The tubes were split into 600 µL aliquots with 500 µL chloroform added and mixed by inversion for 30 min followed by ethanol precipitation as previously described, then resuspended in nuclease-free water.

### Illumina library preparation and short-read sequencing

For each isolate, 1 µg of DNA was sheared to 500 bp fragments with the Covaris M220 Focused Ultrasonicator (Covaris, Woburn, MA, USA) and cleaned with PCRclean DX beads (Aline Biosciences, Waltham, MA, USA) using a 1:1.5 sample-to-bead ratio. Shotgun WGS libraries were prepared with the KAPA Hyper Prep Kit (Roche, Basel, Switzerland) following the manufacturer’s specifications. A double-sided size selection was performed with PCRclean DX beads (0.45–0.65×) to target a final insert size of 500 bp. Paired-end 2 × 150 sequencing on an Illumina NovaSeq was completed at the University of Connecticut Center for Genomic Innovation (https://cgi.uconn.edu/).

### Oxford Nanopore library preparation and long-read sequencing

DNA was enriched for fragments >1.5 kbp using SPRI beads in a modified buffer (10 mM Tris-HCl, 1 mM EDTA pH 8, 1.6 M NaCl, and 11% PEG 8000) with a ratio of 0.8× bead-to-sample volume. Approximately 1 µg of high molecular weight DNA for each sample was used for library prep following the Oxford Nanopore protocol for Native Barcoding Genomic DNA with the Ligation Sequencing Kit (SQK-LSK109) and Native Barcoding Expansion Kit (EXP-NBD104). Libraries were sequenced on a MinION R9.4.1 flow cell, and basecalling was performed with Guppy v6.4.8 using the fast basecalling algorithm. The resulting sequences were quality filtered using the NanoPack programs’ nanofilt (79), retaining sequences greater than 150 bp with a FAST5 q score of 10.

### Genome assembly, annotation, and taxonomy

Preliminary assemblies were generated using the following assemblers with default parameters: MaSuRCA v4.1.0 hybrid assembler (80); SPAdes v3.15.5 hybrid assembler (81, 82); Canu v2.1.1 (83) with POLCA short-read polishing (84); Flye v2.9.2 long-read assembler (85); Miniasm v0.3 long-read assembler (86) with Minipolish v0.1.3 short-read polishing (87); and Raven v1.8.1 (88). Trycycler v0.5.4 (89) used the six preliminary assemblies as input to generate a consensus genome assembly for each isolate. Clusters of contigs were manually curated by discarding clusters with three or fewer contigs and then reconciled into circular elements. When reconciliation failed, contigs required manual trimming, particularly in instances where contigs within a cluster were approximately double the size of contigs from the same cluster. Curation was performed by searching for sequences that appear in the beginning or end that are repeated within the contig and deleting all bases after or before the repeated sequence, respectively. Trycycler then used Muscle (90) to generate a multiple sequence alignment of each cluster for which the entire read set can be aligned to generate a consensus sequence for each replicon. Illumina short reads were aligned to the Trycycler consensus sequence with BWA v0.7.17 (59) and polished with PolyPolish v0.5.0 (60) and POLCA (84). The resulting consensus genomes for each isolate were assessed for completion and contamination with BUSCO v5.4.5 (91) with the bacteria_odb10 lineage and CheckM v1.2.2 (92).

Annotations were performed with Prokka v1.14.5 (93) and eggNOG-mapper v2.1.9 (94). The resulting KEGG orthology terms were aggregated by their functions, and counts were visualized with the “pheatmap” v1.0.12 package in R. Genome-based taxonomy was performed with the largest replicon (containing the 16S rRNA genes) using the Type (Strain) Genome Server (TYGS; 95) to predict the closest type-strain by providing a dDDH, where <70% dDDH predicts a novel species. Information on nomenclature and associated taxonomic literature was provided by the List of Prokaryotic names with Standing in Nomenclature (LPSN; [95]).

The putatively novel species, *Thalassobaculum* sp. OXR-137, *Sulfitobacter* sp. OXR-159, *Idiomarina* sp. OXR-189, and *Christiangramia* sp. OXR-203 were further compared to their closest predicted type-strains and a complete genome of a different species from the type-strain with high 16S rRNA sequence identity from GenBank. The dDDH between the curated genomes and the novel isolates was calculated with the Genome-to-Genome Distance Calculator v3.0 (95) and the ANI was calculated with FastANI v1.3.3 (29). In addition, BLASTx was used to assess protein homology in genome comparisons, using an *E* value cutoff of 1.0e^−20^. Synteny analysis was performed using MCscan v1.3.8 (96), and non-syntenic GIs were identified as 10 or more non-syntenic genes separated by fewer than 10 syntenic genes. GI annotations and functions were supplemented with NCBI’s PGAP pipeline (97) and GhostKoala against the “genus_prokaryotes + virus” database, respectively (98). To putatively explain the occurrence of GIs, HGT events were predicted with Alien Hunter v1.7 (32), MGEs were predicted with mobileOG-db v1.6 (33), and prophage elements were predicted with Phigaro v2.3.0 (34) and VirSorter2 v2.2.4 (35) through the Proksee webtool (99).

### Biosynthetic gene cluster and functional analysis

BGCs were first predicted using DeepBGC v0.1.23 (100) and the resulting JSON file was uploaded with its respective genome FASTA file to the antiSMASH v7.0.1 webserver (101). The resulting analysis was then compiled into a table using antismash_coverter.py (https://github.com/sandragodinhosilva/bgc-analysis/tree/main) and plotted in R with ggplot2.

### Transcriptional activity within the vent plume

The activity of the isolates within the plume was assessed by aligning metatranscriptomic reads from Polinski et al. (15) to each genome with Bowtie2 (102), and count tables generated with RSEM v1.3.3 (103). Resulting count tables were evaluated for differential expression with DESeq2 (104) by comparing samples taken within the plume against samples taken outside of the plume. To assess overall metabolic activity, normalized read counts (TPM) for all genes identified with KEGG orthology (KO) terms were first binned by the KO term and then KEGG pathway to evaluate pathway-level responses within the plume (summed normalized read counts of samples within the plume) versus outside the plume (summed normalized read counts of samples out of the plume). The resulting table for each genome was then combined and ordered by abundance, and the top 30 pathways receiving the most counts were visualized with the “pheatmap” v1.0.12 package in R.

## Data Availability

Complete assembled genomes and raw sequencing reads were submitted to NCBI under project accession number PRJNA1046213. Relevant code and data products produced as part of this study can be found at https://osf.io/8czpm/ and https://github.com/smajor01/Moytirra-Hydrothermal-Vent-Bacterial-Genomes/tree/main.

## SUPPLEMENTAL MATERIAL

Supplemental Figures aem.02593-24-s0001.docx Figures S1 and S2.

Supplemental Tables aem.02593-24-s0002.xlsx Tables S1 to S14.

ASM does not own the copyrights to Supplemental Material that may be linked to, or accessed through, an article. The authors have granted ASM a non-exclusive, world-wide license to publish the Supplemental Material files. Please contact the corresponding author directly for reuse.
