# Supplementary material for: Novel and diverse features identified in the genomes of bacteria isolated from a hydrothermal vent plume
Source: Appl Environ Microbiol. 2025 Mar 31;91(4):e02593-24. doi: 10.1128/aem.02593-24 (PMC12016528; doi:10.1128/aem.02593-24)
Supplement: Supplemental Figures — Figures S1 and S2. [file aem.02593-24-s0001.docx]

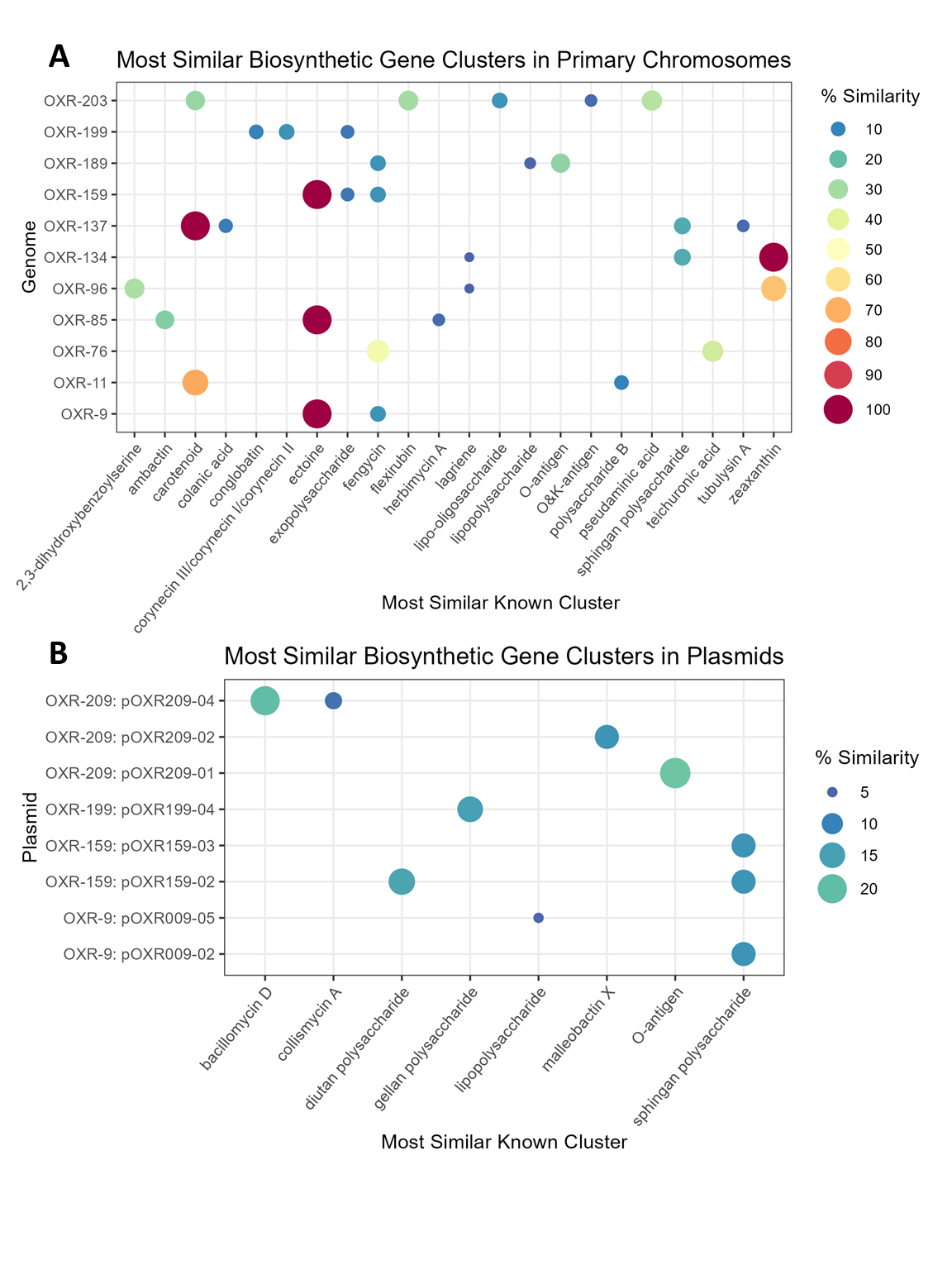


**Figure S1** The percent similarity of known biosynthetic gene clusters predicted to be in; A) the primary chromosomes; B) the plasmids.


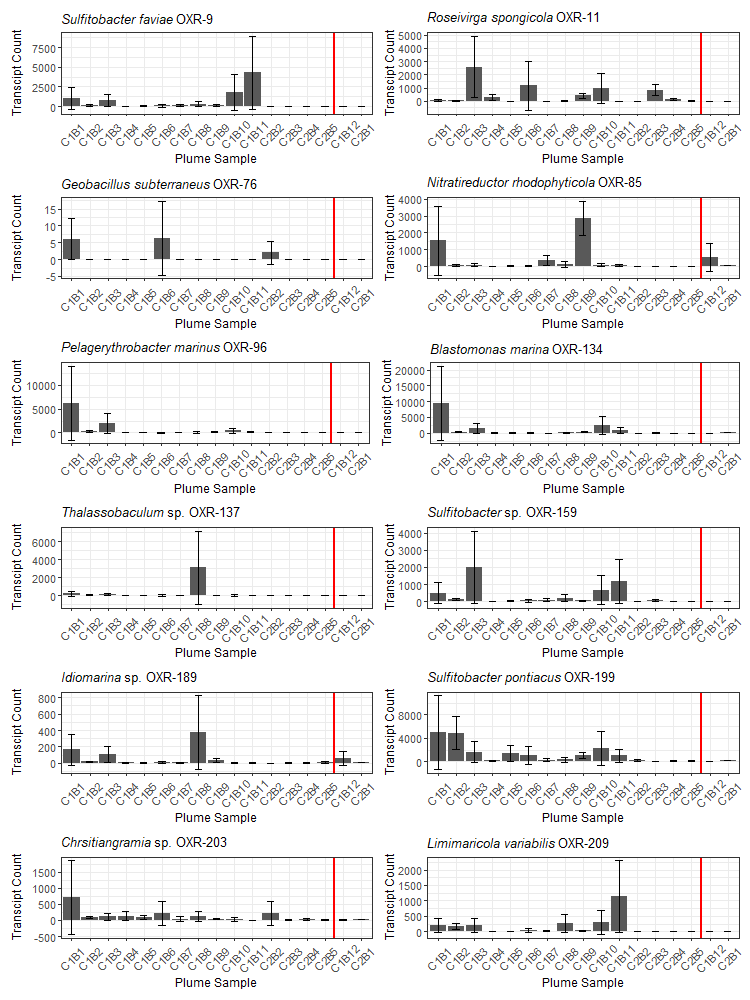


**Figure S2** Mapped read counts for each isolate based on metatranscrptomic data collected along the hydrothermal vent plume (Polinski et al. 2023). The red bar separates samples collected within the plume (left) vs out of the plume (right). Error bars represent the standard deviation of 3 replicate samples.
